# The cost-effectiveness of prostate cancer screening using the Stockholm3 test

**DOI:** 10.1371/journal.pone.0246674

**Published:** 2021-02-25

**Authors:** Andreas A. Karlsson, Shuang Hao, Alexandra Jauhiainen, K. Miriam Elfström, Lars Egevad, Tobias Nordström, Emelie Heintz, Mark S. Clements

**Affiliations:** 1 Department of Medical Epidemiology and Biostatistics, Karolinska Institutet, Stockholm, Sweden; 2 Swedish eScience Research Centre, Stockholm, Sweden; 3 BioPharma Early Biometrics and Statistical Innovation, Data Science & AI, BioPharmaceuticals R&D, AstraZeneca, Gothenburg, Sweden; 4 Department of Laboratory Medicine, Karolinska University Hospital, Stockholm, Sweden; 5 Department of Oncology-Pathology, Karolinska University Hospital, Stockholm, Sweden; 6 Department of Clinical Sciences, Danderyd Hospital, Stockholm, Sweden; 7 Department of Learning, Informatics, Management and Ethics, Karolinska Institutet, Stockholm, Sweden; University of Toronto, CANADA

## Abstract

**Objectives:**

The European Randomized Study of Screening for Prostate Cancer found that prostate-specific antigen (PSA) screening reduced prostate cancer mortality, however the costs and harms from screening may outweigh any mortality reduction. Compared with screening using the PSA test alone, using the Stockholm3 Model (S3M) as a reflex test for PSA ≥ 1 ng/mL has the same sensitivity for Gleason score ≥ 7 cancers while the relative positive fractions for Gleason score 6 cancers and no cancer were 0.83 and 0.56, respectively. The cost-effectiveness of the S3M test has not previously been assessed.

**Methods:**

We undertook a cost-effectiveness analysis from a lifetime societal perspective. Using a microsimulation model, we simulated for: (i) no prostate cancer screening; (ii) screening using the PSA test; and (iii) screening using the S3M test as a reflex test for PSA values ≥ 1, 1.5 and 2 ng/mL. Screening strategies included quadrennial re-testing for ages 55–69 years performed by a general practitioner. Discounted costs, quality-adjusted life-years (QALYs) and incremental cost-effectiveness ratios (ICERs) were calculated.

**Results:**

Comparing S3M with a reflex threshold of 2 ng/mL with screening using the PSA test, S3M had increased effectiveness, reduced lifetime biopsies by 30%, and increased societal costs by 0.4%. Relative to the PSA test, the S3M reflex thresholds of 1, 1.5 and 2 ng/mL had ICERs of 170,000, 60,000 and 6,000 EUR/QALY, respectively. The S3M test was more cost-effective at higher biopsy costs.

**Conclusions:**

Prostate cancer screening using the S3M test for men with an initial PSA ≥ 2.0 ng/mL was cost-effective compared with screening using the PSA test alone.

## Introduction

Prostate cancer is the most common male cancer diagnosed, the third most common cause of male cancer death, and the fourth highest cost by cancer site in Europe [1, 2]. The European Randomised Study of Screening for Prostate Cancer (ERSPC; ISRCTN49127736) found that four-yearly screening for prostate cancer, using the prostate-specific antigen (PSA) test, for ages 55–69 years increased incidence by 41% and reduced mortality by 20% over 16 years [3]. The PSA test is inexpensive but has diagnostic limitations that lead to unnecessary biopsies, over-diagnosis, over-treatment and increased costs [3–6]. We use the term “screening” in a manner that includes both organised screening and opportunistic testing.

Some commentators take the view that the costs and harms from prostate cancer screening outweigh the health benefits from early detection. A contrasting view is that prostate cancer screening has become so widely accepted that we should consider harm and cost reduction strategies for prostate cancer screening. Reflecting these two views, the US Preventive Services Task Force recommended against PSA screening in 2012 [7], which was followed by a limited decline in PSA screening in the United States, and then the Task Force changed their recommendation to shared decision-making in 2018 [8]. One consequence of this debate is that few healthcare systems have organised prostate cancer screening.

To reduce the downstream costs and potential harms associated with PSA screening, a number of new screening tests and risk calculators have been developed, including the 4K score, the Prostate Health Index, PCA3 (a urine-based test) and the ERSPC risk calculators [9], however there have been comparatively few economic evaluations [10–12].

**Fig 1:**
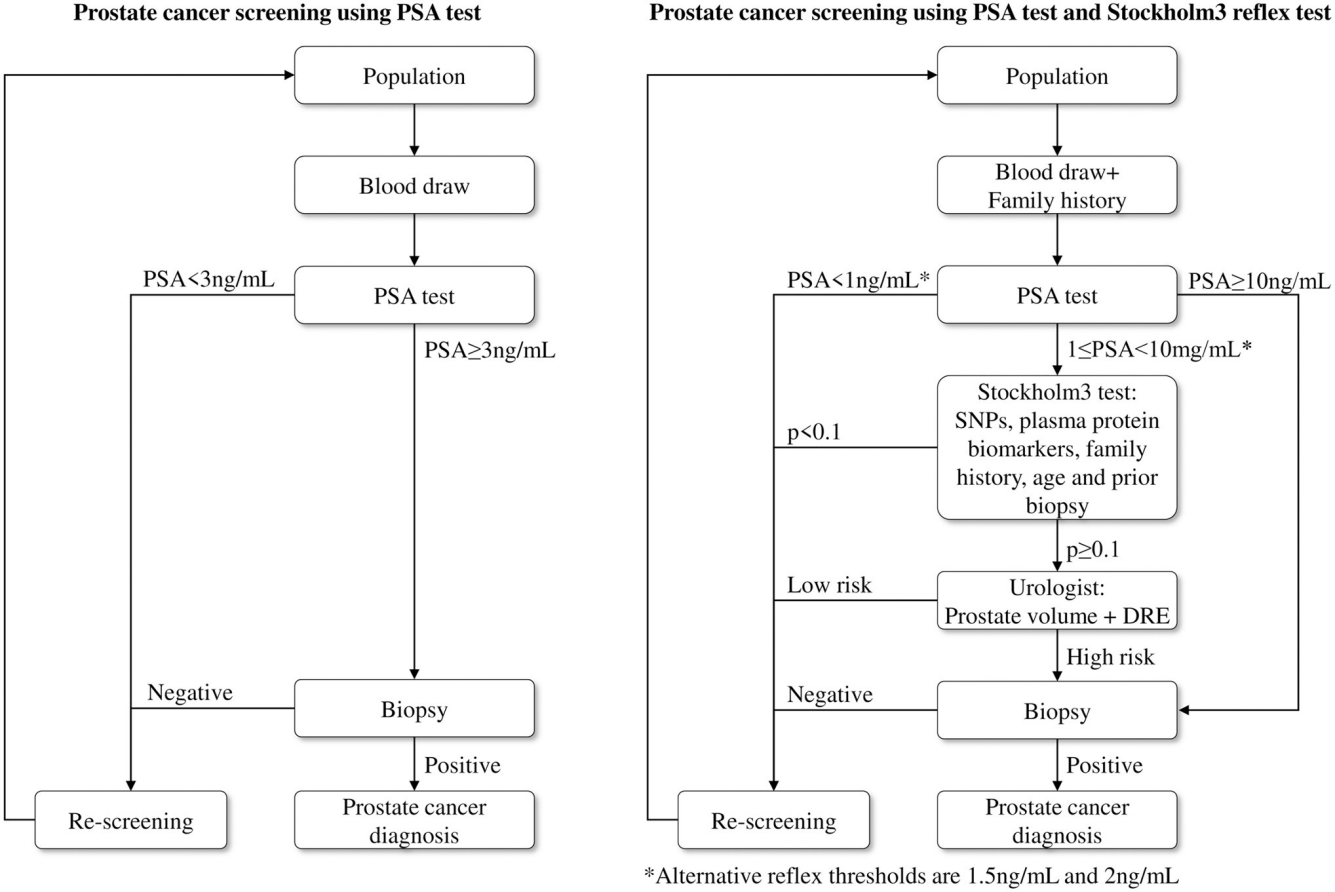
Prostate cancer screening interventions using PSA test and the S3M reflex test.

A large prostate cancer diagnostic trial in Stockholm (Stockholm3; ISRCTN84445406) combined blood analyses for proteins, a genetic risk score and other clinical variables [13]. The trial included prospective recruitment of men from the general population to assess their disease status using a paired screen-positive design. The Stockholm3 Model (S3M) included the following steps: (1) an initial blood draw and a short questionnaire; (2) laboratory evaluation of the PSA test; (3a) for men with 1 ≤ PSA < 10 ng/mL, a S3M risk prediction for Gleason score 7 based on a genetic risk score based on a single nucleotide polymorphism (SNP) chip, five plasma protein biomarkers, together with self-reported age, family history and any previous negative biopsies; (3b) for men with PSA 10 ng/mL, referral to a urologist for a biopsy; (4) for men with an S3M risk above 10%, referral to a urologist who would decide to undertake a biopsy based on the man’s prostate volume measured with trans-rectal ultrasound and the results from a digital rectal exam; and (5) for men with an S3M risk below 10% and a PSA test below 10 ng/mL, referral to re-screening (Fig 1, right panel). Screening with the PSA test included the following steps: (1) an initial blood draw and a short questionnaire; (2) laboratory evaluation of the PSA test; (3) prostate biopsy for men with PSA ≥ 3ng/mL; (4) for men with PSA<3ng/mL or negative biopsy, referral to re-screening (Fig 1, left panel). A pathologist classifies the biopsy as either benign or cancer, with cancer classified histo-pathologically as Gleason score 6, which associated with a good prognosis, and Gleason score 7 or over, which is associated with poorer prognosis. Compared with men who had PSA between 3 and 10 ng/mL, the S3M test with a reflex threshold of 1 ng/mL had a relative positive fraction of 0.56 (95% confidence interval (CI): 0.46–0.65) for no cancer, with a relative positive fraction of 0.83 (95% CI: 0.74–0.93) for Gleason score 6 cancers, while maintaining the sensitivity for Gleason score 7 cancers (that is, a relative positive fraction of 1). One minus the relative positive fraction can be interpreted as the relative change in the number of biopsies. The test characteristics of the S3M test are currently being evaluated in other populations. Recently, the prediction algorithm was updated to use the full study dataset, with the inclusion of a high-risk SNP and removal of one protein biomarker [14]. Nordström and colleagues found that changing the PSA reflex threshold for the S3M test from 1.0 ng/mL to 1.5 ng/mL reduced the number of S3M tests by a third [15]. As a further cost-reduction strategy, we were also interested in a reflex threshold of 2 ng/mL. In a screening context, using S3M as a reflex test may be associated with higher screening costs. However, the reduction in biopsies and over-treatment are expected to improve the health-related quality of life and reduce costs. The cost-effectiveness of the S3M test has not previously been assessed.

Our aim is to assess the long-term health effects and cost-effectiveness of PSA screening with S3M used as a reflex test above a PSA of 1, 1.5 or 2 ng/mL compared with no prostate cancer screening or PSA screening alone.

## Methods

To assess the cost-effectiveness of five screening interventions for prostate cancer, we simulated the health effects and costs of each intervention using the Swedish “Prostata” microsimulation model [16, 17]. The microsimulation model combined a natural history model with the test characteristics and screening interventions. The five screening interventions were no screening, PSA screening, and S3M screening at three different reflex thresholds (the interventions are described in more detail under the sub-section on Screening interventions). The interventions combined the screening age range, re-screening interval and screening effectiveness from ERSPC with test characteristics from Stockholm3. The natural history model for prostate cancer onset, progression, and survival was calibrated to data from the Stockholm PSA and Biopsy Register (SPBR) [18], the Swedish National Prostate Cancer Register [19] and ERSPC. The cost-effectiveness was analysed from a lifetime societal perspective. The main outcomes were the incremental cost-effectiveness ratios (ICERs), defined as the costs per quality-adjusted life years (QALYs) gained. As per Swedish government recommendations, both costs and QALYs were discounted at 3% per year [20].

### Ethics

The Stockholm3 Study was approved by the Ethical Review Board, Stockholm (dnr 2012/438-31/3). The registration number for the Stockholm3 Study is NCT03639649/ ISRCTN84445406. All study participants have given written informed consent to publish these case details. The Stockholm PSA and Biopsy Register was approved by the Ethical Review Board, Stockholm (dnr 2012/438-31/3, dnr 2016/620-32) and the data were analysed anonymously.

### Screening interventions

The five screening interventions were no screening and quadrennial screening for men aged 55-69 years with either the PSA test alone or with an initial PSA test and a reflex S3M test for PSA values above 1, 1.5 and 2 ng/mL, respectively (see Fig 1). For the PSA screening intervention, we used a threshold of 3 ng/mL for referral to a urologist. For the reflex S3M screening interventions, men with either a PSA of 10 ng/mL or a PSA between the reflex threshold and 10 ng/mL with a positive reflex S3M test were referred to a urologist. Re-screening after age 70 years was only for clinical follow-up of test positive and biopsy negative men. We assumed that screening would be administered through general practitioners.

### Simulation model

**Fig 2:**
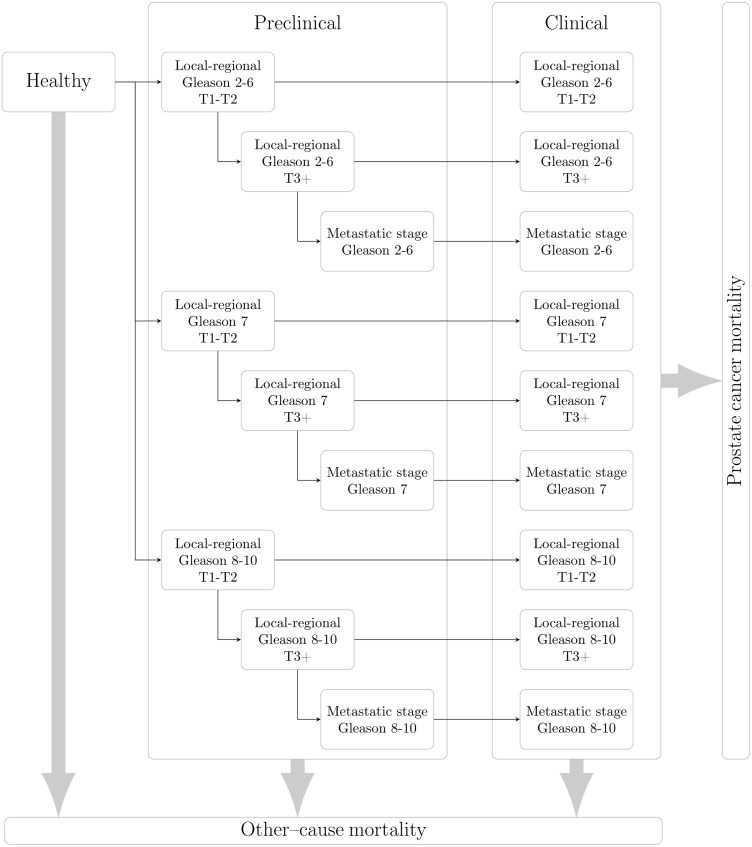
Schematic of the prostate cancer natural history model. Individuals are assumed to be disease-free at age 35 years. They may progress to preclinical cancer states with a fixed Gleason score, with progression by T-stage and to metastatic cancer. Preclinical cancers may be diagnosed from nine different states, with survival from prostate cancer death evaluated from the possibly counterfactual time of clinical diagnosis. Death due to other causes is represented as a competing event.

The Prostata model extended an earlier American model of prostate cancer [21]. The model simulated individual stochastic life histories from birth including PSA values, prostate cancer onset, metastatic progression, diagnosis, treatment and survival. Using data from the SPBR, we modelled for PSA screening, biopsy compliance and prostate cancer treatment, including active surveillance, radical prostatectomy and radiation therapy. Within a stochastic simulation context, we calculated the mean quality-adjusted life-years and mean costs across individuals for each screening intervention. The model is open source and available as an R package (http://github.com/mclements/prostata). The natural history model includes pre-clinical progression for T- and M-stage for a given Gleason score prior to diagnosis (see Fig 2). The screening and treatment pathways are described in S3 Fig in S1 Text. For further details, including extensive model calibration and validation using data from Sweden and Europe, see Karlsson et al [17].

### PSA and S3M test characteristics

A positive S3M test was defined as one having a PSA value above the reflex threshold and a risk prediction above 10%. S3M test characteristics were defined based on the proportion of men for whom an S3M test was positive with PSA values between the reflex threshold and 10 ng/mL divided by the proportion with PSA 3–10 ng/mL. The three health or disease states, represented by *D*, included no cancer, Gleason score 6 cancers and Gleason ≥ 7 cancers. The S3M reflex threshold was represented by *α*. The relative positive fraction *r*(*α*|*D*) for a threshold *α* for a given state *D* was defined as [22]

$$\begin{array}{c} r(\alpha |D)=\frac{\text{Pr}({\text{S3M}}^{+}|D,\alpha \leq \text{PSA}<10)}{\text{Pr}(3\leq \text{PSA}<10|D,\alpha \leq \text{PSA}<10)} \end{array}$$

These relative positive fractions generalised the relative true- and false-positive fractions for three health or disease states. Values for *r*(*α*|*D*) were estimated from the Stockholm3 trial (see Table 1 Part A). Test characteristics for a reflex threshold of 1 ng/mL were described in [13]. For example, *r*(*α* = 1|*D* = (Gleason ≥ 7)) = 1 for S3M having the same sensitivity as PSA for Gleason score ≥ 7 cancers with a reflex threshold of *α* = 1 ng/mL. To calculate the reflex thresholds of 1.5 and 2 ng/mL, we re-analysed the Stockholm3 trial assuming the same S3M test threshold for referral to a urologist.

**Table 1:** Input parameters.

**Part A. Test characteristics (relative positive fractions compared with 3 ≤ PSA < 10) for reflex thresholds 1ng/mL, 1.5ng/mL and 2ng/mL**						
Disease state	Reflex test threshold					
	1ng/mL	(95% CI)	1.5ng/mL	(95% CI)	2ng/mL	(95% CI)
Benign	0.56	(0.46–0.65)	0.52	(0.44–0.60)	0.48	(0.40–0.55)
GS = 6	0.83	(0.74–0.93)	0.79	(0.72–0.87)	0.72	(0.66–0.80)
GS≥7	1.00	–	0.98	(0.94–1.03)	0.95	(0.91–0.99)
CI: Confidence interval; GS: Gleason score; Source: [13]						

Part B. Health state values			
Health state	Health state value	Duration	Unit
PSA test	0.99	1	Week
Biopsy	0.90	3	Week
Cancer diagnosis	0.80	1	Month
Radical prostatectomy (part1)	0.67	2	Month
Radical prostatectomy (part2)	0.77	10	Month
Radiation therapy (part1)	0.73	2	Month
Radiation therapy (part2)	0.78	10	Month
Active surveillance	0.97	7	Year
Post recovery period	0.95	9	Year
Palliative therapy	0.60	30	Month
Terminal illness	0.40	6	Month
PSA: Prostate-specific antigen; Source: [24]			

Part C. Costs and productivity losses for prostate cancer screening, diagnosis, management and treatment				
Cost item	Unit cost (€)	Production loss item	Duration	Unit
PSA test (including GP visit)	60	PSA test (including GP visit)	2	Hour
S3M test (including GP visit)	255	S3M test (including GP visit)	2	Hour
Biopsy (excluding assessment)	403	Biopsy (excluding assessment)	2	Hour
Urology assessment	153	Urology assessment	2	Hour
Radical prostatectomy	9 443	Radical prostatectomy	6	Week
Radiation therapy	9 018	Radiation therapy	8	Week
Active surveillance–yearly	362	Metastatic cancer (excluding	6	Month
Active surveillance–Single MRI	263	terminal illness)		
Post-treatment follow-up–yearly	74	Terminal illness	6	Month
Advanced disease	42 920			
GP: General practitioner; MRI: Magnetic resonance imaging; PSA: Prostate-specific antigen; S3M: Stockholm3 test; Source: [25]				

Simulating from the Stockholm3 Trial, we found test characteristic thresholds on the PSA scale with corresponding test characteristics of the S3M test. We defined *τ*(*α*|*D*) as the PSA threshold for a given underlying health or disease state. For each state *D*, we chose the test characteristics threshold so that the number of men with PSA at or above *τ*(*α*|*D*) was equal to the number of men who had a PSA between 3 and 10 ng/mL times the relative fraction *r*(*α*|*D*), such that

$$\begin{array}{cc} \text{Pr}(\text{PSA}\geq \tau (\alpha |D)|D,\alpha \leq \text{PSA}<10) & =\text{Pr}({\text{S3M}}^{+}|D,\alpha \leq \text{PSA}<10)\\ & =r(\alpha |D)\text{Pr}(3\leq \text{PSA}<10|D,\alpha \leq \text{PSA}<10) \end{array}$$

For example, for a reflex threshold of 1 ng/mL and a Gleason score of 6, the S3M test characteristic had a relative positive fraction of 0.83 (that is, a 17% decrease in GS 6 biopsies), and the corresponding PSA test characteristic was 3.76 ng/mL. Men with a PSA below the test characteristics threshold were assumed to be S3M negative. We assumed that the new test would have similar prognostic characteristics as the PSA test.

### Clinical and epidemiological outcomes

The modelled outcomes include numbers of screening tests, biopsies, prostate cancer incidence, prostate cancer death, over-diagnosis (defined as the lifetime risk of a prostate cancer diagnosis for individuals who would never have had clinical symptoms), and life expectancy. A “screen-detected prostate cancer” was defined as an asymptomatic cancer detected through an investigation initiated by screening, rather than due to symptoms.

### Quality-adjusted life-years (QALYs)

To measure effectiveness in the cost-effectiveness analysis, QALYs were calculated for each screening intervention. These were calculated by multiplying the health-related quality of life in each health state (the health state value) with the time in that health state. The overall health state values were calculated as the product of the health state value of the general population [23] with those for the modelled health states (see Table 1 Part B for details and Heijnsdijk et al [24]).

### Costs

For our study, both direct and indirect costs were considered. Direct costs included costs of visits to a general practitioner, screening test analysis, diagnosis, prostate cancer treatment, clinical follow-up, palliative treatment and terminal care. All costs, except those specific to S3M, were reported in a health economic assessment of organised prostate cancer screening using the PSA test in Sweden [25]. The total cost for a PSA test was €58, including test sampling (€30), PSA analysis (€4) and 20% of a primary care visit (net €26). The total cost for undertaking an S3M test was €255, including the same costs for test sampling and primary care as for PSA alone and the cost for the S3M test itself at €196 (77% of the total).

For men who had their prostate cancer diagnosed because of symptoms, we assumed that on average there would be two diagnostic biopsies (see the S1 Text).

The direct costs were calculated by summing the unit costs for each simulated event. Indirect costs were calculated based on productivity losses, which were estimated using the time lost for specific events multiplied by the equivalent age-specific salary of work for males and females combined [26]. Estimates of the work time lost due to screening, screening and treatment, together with age-specific mean salaries, including social fees, were reported by the Swedish National Board of Health and Welfare (NBHW) [25]. The detailed costs are given in Table 1 Part C. Costs were measured in Swedish kronor (SEK) in 2016 and converted to Euros in the Euro area in 2019, adjusting for differences in price levels using purchasing power parities at €0.0851/SEK [27].

The NBHW have defined categories for the cost per QALY gained, with costs under 100,000 SEK (€8,300) described as low, 100,000–500,000 SEK (€8,300–41,600) as moderate, 500,000–1 million SEK (€41,600–83,300) as high, and costs over 1 million SEK (€83,300) per QALY gained as being very high [28]. We defined an intervention A as being dominant over an intervention B if intervention A had higher QALYs and lower costs than intervention B.

### Sensitivity analyses

Sensitivity analyses were reported (a) by specific model components using one-way sensitivity analyses and (b) for multiple parameters using a probabilistic analysis with cost-effectiveness acceptability curves. For one-way sensitivity analyses, we investigated: (a) the cost of the S3M test; (b) the discount rate between 0% and 5%; (c) the S3M test characteristics between the upper and lower CI; (d) health state value decrements for the health states (±20%), excluding the background age-specific health state values; (e) cost of a biopsy episode, including the urological consultation, biopsy procedure and pathology (baseline €560, with a range of €330 to €880); and (f) all indirect and direct costs (±20%). For (e), the upper bound for the cost of a prostate biopsy was based on the 2017 price list for Stockholm region and the lower bound was symmetric on a multiplicative scale.

The probabilistic analysis included estimated natural history parameters, S3M test performance, costs and health state value decrements. We assumed that the natural history parameters were multivariate normal. The S3M test performance was assumed to be normally distributed. The costs for biopsy and urology assessment were assumed to be log-normal with a mean of €560 (95% CI 330-880). The other costs and the health state value decrements where sampled from triangular distributions with extremes 20%. The model was evaluated with new parameter combinations 500 times. Note that the number of parameter combinations was constrained by computational resources. The interventions were compared using cost-effectiveness acceptability curves, calculating the proportion of simulations where an intervention was cost-effective compared with a reference intervention at a given willingness-to-pay threshold.

## Results

### Main findings

Predicted outcomes under the five interventions are summarised in Table 2. For 10,000 men who were not screened, approximately 1487 men would be diagnosed with prostate cancer and 502 men would die due to the disease.

**Table 2:** Predicted effects for no screening, PSA screening and S3M screening at 4-year intervals for the ages 55 to 69 years. The effects are presented as clinical events per 10,000 men, cost as Euros per man and cost-effectiveness as €/QALY. The four right most columns contrasts the clinical and costs as differences and cost-effectiveness as incremental cost-effectiveness ratios.

Lifetime predictions	No screening	PSA	S3M_1+_	S3M_1.5+_	S3M_2+_	PSA-no screening	S3M_1+_-PSA	S3M_1.5+_-PSA	S3M_2+_-PSA
**Outcomes per 10,000 men**						**Differences**			
Screening tests	0	35,804	34,943	34,864	34,792	35,804	-861	-939	-1,011
Biopsies	2,789	7,947	5,903	5,711	5,525	5,158	-2,044	-2,235	-2,420
Negative biopsies	1,302	6,339	4,307	4,119	3,937	5,037	-2,031	-2,220	-2,401
Diagnosed cancers	1,487	1,608	1,596	1,593	1,588	121	-12	-15	-19
Screen-detected cancers	0	534	509	503	494	534	-25	-31	-40
Overdiagnosed cancers	0	121	109	106	102	121	-12	-15	-19
Prostate cancer death	502	430	433	433	434	-71	2	3	4
LY	794,308	794,960	794,943	794,939	794,930	652	-18	-22	-30
QALYs	694,959	695,325	695,330	695,329	695,327	366	4	4	1
**Costs (€) per man, undiscounted**									
Screening	0	214	518	429	378	214	304	216	164
Diagnosis	155	445	338	327	316	290	-108	-119	-129
Treatment	721	1,015	996	991	984	294	-20	-24	-31
Advanced disease	2,153	1,847	1,857	1,860	1,865	-306	11	13	18
Total direct costs	3,029	3,521	3,708	3,607	3,542	492	187	86	21
Loss in productivity	91	168	164	163	161	77	-4	-5	-7
Total societal costs	3,120	3,689	3,872	3,770	3,703	569	183	81	14
**€/QALY, undiscounted**						**ICER**			
Health sector						13,459	438,013	204,444	158,683
Societal						15,551	428,888	192,598	108,740
**€/QALY, 3% discounted**									
Health sector						48,392	177,751	68,449	11,262
Societal						54,918	173,921	64,131	5,663

**S3M_1+_** a S3M reflex test for PSA values between 1 and 10 ng/mL [13].

**S3M_1.5+_** a S3M reflex test for PSA values between 1.5 and 10 ng/mL as per clinical practice [29].

**S3M_2+_** a S3M reflex test for PSA values between 2 and 10 ng/mL.

For quadrennial screening of men aged 55–69 years with a positive PSA test at ≥ 3 ng/mL, there were 121 more prostate cancer diagnoses per 10,000 men. PSA screening decreased the lifetime risk of prostate cancer death, with 71 fewer deaths per 10,000 men, with an increase of 652 life-years and 366 QALYs per 10,000 men. The change in undiscounted life-years is primarily affected by differential prostate cancer mortality, while the change in QALYs is a combination of differential mortality and a loss of utilities due to over-screening, unnecessary biopsies and over-diagnosis. PSA screening was associated with increased costs for screening (€214/man), diagnostic work-up (€290/man), treatment for localised prostate cancer (€294/man) and productivity losses (€77/man), with lower costs for treatment for advanced prostate cancer (-€306/man). Discounting at 3% per annum, the incremental cost-effectiveness ratio (ICER) from a societal perspective was €54,918/QALY, which represents a high cost per QALY gained in Sweden.

Compared with PSA screening alone, using the S3M test at a reflex threshold of ≥ 1.5 ng/mL was predicted to reduce the lifetime number of screening tests by 3%, diagnostic biopsies by 28% and prostate cancers diagnosed during a man’s lifetime by 1%, with 4 additional QALYs and, conversely, 3 additional prostate cancer deaths per 10,000. Comparing costs, S3M was associated with a 101% increase in screening costs, 1% increase in treatment costs for advanced prostate cancer, 27% decrease in costs for diagnostic work-up, 2% decrease in treatment costs for localised cancer and a 3% decrease in productivity losses, leading to an overall increase of 2% in total societal costs, or €81/man,. Taking a societal perspective, the discounted ICER was €64,131/QALY, which is a high cost per QALY gained in Sweden. The high costs were due to a marked increase in screening costs, only partially offset by the decrease in costs due to diagnostic work-up.

Compared with PSA screening alone, S3M reflex thresholds of 1 and 2 ng/mL were associated with a 26% and 30% reduction in the number of biopsies and a 24% and 29% reduction in costs for diagnosis, respectively. The difference in screening costs were 142% and 77% of the screening costs for PSA, respectively; and smaller changes for the other outcomes and costs associated with a reflex threshold of 1.5 ng/mL (Table 2). Relative to PSA screening alone, the total societal costs were lower for higher reflex thresholds, and the discounted ICERs were substantially lower: for the 1 ng/mL reflex threshold, the ICER was €173,921/QALY, which is a very high cost per QALY gained in Sweden, while for the 2 ng/mL reflex threshold, the ICER was €5,663/QALY, which is a low cost per QALY gained in Sweden. Compared with no screening, S3M at reflex thresholds of 1, 1.5 and 2 ng/mL were associated with ICERs of €64,021/QALY, €55,707/QALY and €50,577/QALY, respectively. For incremental cost-effectiveness compared with no screening, S3M with a reflex threshold of 2 ng/mL had an ICER 9% lower than the ICER for PSA screening alone.

**Fig 3:**
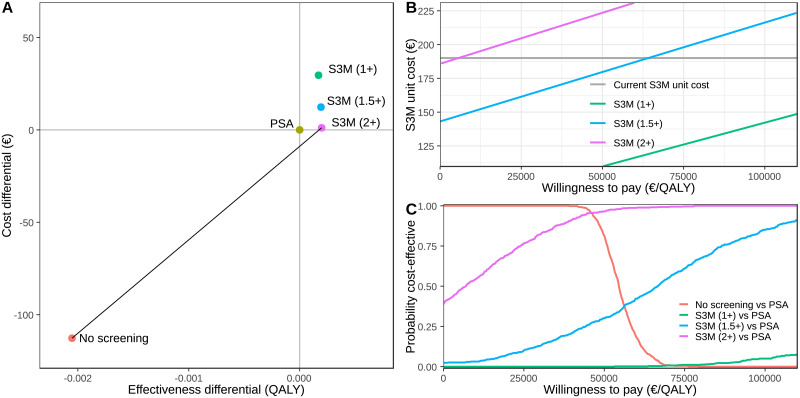
Panel (A) shows the cost-effectiveness plane comparing no screening, PSA screening and S3M screening with reflex thresholds at 1 ng/mL, 1.5 ng/mL and 2 ng/mL. The effectiveness and costs are incremental relative to PSA screening and discounted at 3% per annum. Panel (B) shows the ICER (€/QALY) for S3M as a reflex for PSA above 1, 1.5 and 2 ng/mL as functions of the unit cost of S3M. The cost-effectiveness ratios are incremental to PSA screening alone and are discounted at 3% per annum. The current S3M unit cost, of €196, is shown as the grey line. Finally, Panel (C) shows the probability that an intervention is more cost-effective than PSA screening under parameter uncertainty for a specific willingness to pay threshold.

The discounted cost-efficiency frontier for the five interventions is shown in Fig 3A. The main change in effectiveness was the shift from no screening to screening. The cost-efficiency frontier was between no screening and S3M with a reflex threshold of 2 ng/mL, where PSA screening alone was extended dominated by the S3M reflex threshold of 2 ng/mL. The S3M reflex 2 ng/mL threshold dominated the S3M reflex thresholds for 1 and 1.5 ng/mL, with very similar effectiveness and lower costs.

### One-way sensitivity analyses

We initially investigated the effect of the S3M unit cost on cost-effectiveness compared with PSA alone (Fig 3B). The ICERs increased linearly with an increased S3M unit cost. Using the S3M as a reflex test at 1.0 ng/mL, ICERs of €50,000 and €100,000 per QALY gained were achieved at unit costs of €113 and €147, respectively. Using S3M as reflex test at a PSA of 1.5 ng/mL, ICERs of €50,000 and €100,000 per QALY gained were achieved at unit costs of €185 and €223 respectively. Lastly, S3M as a reflex test at 2.0 ng/mL was dominant over PSA screening alone at a unit cost of €191.

**Fig 4:**
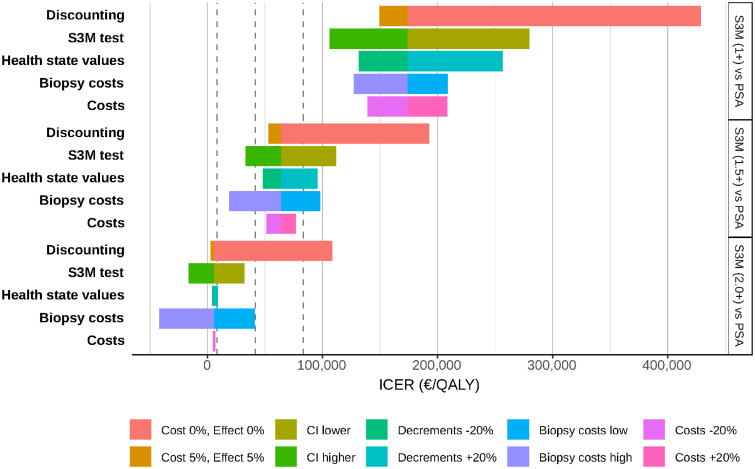
One-way sensitivity analysis showing the effect of no and high discounting rates, the S3M test performance, high and low biopsy costs, 20% variation in all costs and health state value decrements on the cost-effectiveness. From the left, the dashed lines show the limits for low (less than €8,300), moderate (€8,300–41,600), high (€41,600–83,300), and very high costs (over €83,300) for Sweden. The bounds for the biopsy costs, together with the urology assessment, varied between €330 and €880.

We then compared reflex S3M screening at 1.5 ng/mL with PSA screening alone (see Fig 4, middle panel). The costs per QALY gained, for S3M screening, increased with less discounting, higher health state values and higher total costs and poorer S3M test characteristics, while higher biopsy costs led to lower costs per QALY gained. For these sensitivity analyses, a reflex threshold of 1.5 ng/mL would be a moderate to high cost per QALY gained in Sweden compared with PSA screening alone.

For all of the parameters investigated in the sensitivity analyses, S3M at a reflex threshold of 1 ng/mL would be a very high cost per QALY gained compared with PSA screening. Comparing reflex S3M at 2 ng/mL with PSA screening, the reflex S3M test was a high cost per QALY gained when the costs and QALYs were not discounted. For all of the other sensitivity analyses, reflex S3M at 2 ng/mL was either dominant or low to medium cost per QALY gained in Sweden compared with PSA screening alone.

### Probabilistic analysis

The cost-effectiveness acceptability curves (Fig 3C; see also S2 Fig in S1 Text) summarise the uncertainty in the ICER in relation to the willingness to pay. Assuming a willingness to pay of €0/QALY, no screening was more cost-effective than PSA screening with a probability of 100%, and S3M screening with reflex thresholds at 1 ng/mL, 1.5 ng/mL and 2 ng/mL were predicted to be more cost-effective than PSA screening with probabilities of 0%, 2% and 39%, respectively. However with a willingness to pay of €50,000/QALY gained, no screening was more cost-effective than PSA screening with a probability of 71%, whereas S3M screening with reflex threshold at 1 ng/mL, 1.5 ng/mL and 2 ng/mL were predicted to be more cost-effective than PSA screening with probabilities 0%, 31% and 97%, respectively.

## Discussion

Reflex S3M screening maintained the screening benefits of the PSA test and reduced lifetime harms compared with PSA screening alone. Specifically, the reflex S3M test increased discounted quality-adjusted life years and reduced the number of biopsies across a lifetime. The ICERs associated with reflex S3M screening at PSA values of 1, 1.5 and 2 ng/mL were approximately €170,000, €60,000 and €6,000 per QALY gained, which are very high, high and low costs per QALY gained in Sweden, respectively. The S3M test was more cost effective at higher reflex thresholds, at higher biopsy costs and at lower S3M test costs. Compared with no screening, S3M with a reflex threshold of 2 ng/mL had the lowest ICER at 50,577 € per QALY gained, while PSA screening alone had an ICER of 54,918 € per QALY gained.

### Comparison with existing evidence

Our estimates of costs and effectiveness for no screening and PSA screening are broadly similar to estimates from the MISCAN model [4]. Both models were calibrated to the ERSPC trial and used the same health state values [24], while the costs for treatment of advanced cancer were €41,683 for Sweden [25] compared with €12,276 for the Netherlands [30]. The MISCAN model does not explicitly model for the PSA value, requiring stronger assumptions about how the test characteristics vary by screening interventions. The Prostata natural history model was calibrated to contemporary Gleason score-specific incidence and survival, which are critical for any evaluation of the S3M test.

Our cost-effectiveness analysis between the S3M test and PSA is broadly comparable with an evaluation of the Prostate Health Index (PHI) [11]. Note, however, that it is difficult to directly compare the test characteristics for PHI and S3M due to differences in populations and study design.

### Implications for prostate cancer screening

Compared with no screening, the screening strategies had ICERs that are moderate to high cost per QALY gained, which indicate a high cost to society from prostate cancer screening. If we assume pragmatically that prostate cancer screening is unlikely to be eliminated, then there is an urgent need to further reduce the harms and costs associated with prostate cancer screening.

The cost-effectiveness of the S3M test was sensitive to the cost of the S3M test. The cost of the S3M test is expected to decline with greater use, which we predict would lead to reduced costs and harms and maintain the mortality benefits from early detection. Notably, our study does not include a budget impact analysis, which would be important for planning for the introduction of a new screening test [31].

Multi-parametric magnetic resonance imaging (MRI) may further improve specificity while maintaining the sensitivity for advanced prostate cancer [32, 33]. Screening tests that can reduce the proportion of men referred to a urologist, such as the S3M test, may be increasingly cost-effective in this setting. Such screening tests may be based on combinations of clinical variables and samples based on blood or urine. Results from the forthcoming STHLM3-MRI study will assess the Stockholm3 test in combination with MRI (for the protocol, see [34]).

In the future, it would be useful to consider higher PSA thresholds (e.g. 4 ng/mL), higher reflex thresholds (e.g. 3 ng/mL), different screening and re-screening protocols, and to assess the cost-effectiveness of S3M in combination with MRI.

### Limitations

There are several limitations to this analysis. First, cost-effectiveness analyses are generally limited by the validity and uncertainty of the health state values [26]. Second, we modelled for a positive S3M test based on strictly ordered PSA values and assumed that outcomes would be similar for men who were positive for either the PSA or S3M test. These may be reasonable approximations, as PSA is one of the strongest components of the S3M test [13]. Currently, there is little evidence on longer-term outcomes for men with different S3M biomarker values. Third, we used the human capital approach to estimate productivity losses. This approach has been criticised, as it discriminates against individuals who are aged over 65 years. Fourth, the estimated ICERs may not generalise immediately to other populations, as prostate cancer incidence and mortality rates are comparatively high in Sweden and the costs in Sweden may differ from other countries.

### Strengths of our approach

The strengths of our approach include, first, the use of detailed longitudinal data to inform the mechanistic natural history model. Our model improves on an older US model for the distribution of Gleason scores at diagnosis and on Gleason score-specific survival. These are critical parameters for valid modelling of the new prostate cancer tests, where an ideal test would identify as many or more men with advanced prostate cancer (e.g. Gleason score ≥ 7 cancers) and fewer men with smaller, less advanced prostate cancers (e.g. Gleason score 6 cancers) or negative biopsies. Second, our model code is open source and readily accessible. This approach addresses a long-standing criticism of microsimulation models for cancer screening, where there has been an incomplete description of the models. Third, our mechanistic model has good internal validity for comparing prostate cancer screening interventions. Fourth, the screening effectiveness and S3M test characteristics were based on existing study data rather than extrapolations.

### Conclusions

Compared with quadrennial PSA screening, use of the S3M as a reflex test at PSA ≥ 2.0 ng/mL was predicted to result in a low cost per QALY gained in Sweden. However, use of the S3M test at reflex PSA thresholds of 1 and 1.5 ng/mL were predicted to result in very high and high costs per QALY gained, respectively. Lower S3M test costs would further improve the cost-effectiveness of the S3M test. S3M is a cost-effective reflex test that can reduce harms due to prostate cancer screening while maintaining the health benefits from early detection.

## Supporting information

S1 Text Supplementary material. (PDF)
