# Supplementary material for: The cost-effectiveness of prostate cancer screening using the Stockholm3 test
Source: PLoS One. 2021 Feb 25;16(2):e0246674. doi: 10.1371/journal.pone.0246674 (PMC7906342; doi:10.1371/journal.pone.0246674)
Supplement: S1 Text — (PDF) [file pone.0246674.s001.pdf]

The cost-effectiveness of prostate cancer screening using the Stockholm3 test

Andreas Karlsson[1,2], Shuang Hao[1,2], Alexandra Jauhiainen[3], K Miriam Elfström[4], Lars Egevad[5], Tobias Nordström[1,6], Emelie Heintz[7], Mark Clements[1,2*]

1 Department of Medical Epidemiology and Biostatistics, Karolinska Institutet, Stockholm, Sweden
2 Swedish eScience Research Centre, Sweden
3 BioPharma Early Biometrics and Statistical Innovation, Data Science & AI, BioPharmaceuticals R&D, AstraZeneca, Gothenburg, Sweden
4 Department of Laboratory Medicine, Karolinska University Hospital, SE-141 86, Stockholm, Sweden
5 The Department of Oncology-Pathology, Karolinska University Hospital, Z1:00, SE-171 76, Stockholm, Sweden
6 Department of Clinical Sciences, Danderyd Hospital, SE-182 88 Stockholm, Sweden
7 Department of Learning, Informatics, Management and Ethics, Karolinska Institutet, PO Box 281, SE-171 77, Stockholm, Sweden
* mark.clements@ki.se

Supplementary Material

Means costs and QALYs

Mathematically, the mean costs (Cost_k) and QALYs (QALY_k) for intervention k were calculated by

$$\text{Cost}_k = \sum_{i=1}^{n} \frac{1}{n} \int_0^\infty \frac{dC_{ik}(t)}{(1+\delta)^t}, \qquad \text{QALY}_k = \sum_{i=1}^{n} \frac{1}{n} \int_0^\infty \frac{dV_{ik}(t)}{(1+\delta)^t}$$

where i was an index over the simulated individuals, $dC_{ik}(t)$ and $dV_{ik}(t)$ were the cumulative costs and HSVs, respectively, for individual i under intervention k at time t, and δ was the discount rate (e.g. 3%). This formulation allowed for both discrete components (e.g. costs at a point in time) and continuous components (e.g. a fixed HSV over a defined period of time). The interventions were compared using the incremental costs and incremental QALYs.

Number of biopsies for clinically detected prostate cancers

The simulation model did not explicitly model for biopsies for men who had symptoms but who did not have clinically detectable prostate cancer. These biopsies were modelled implicitly by the number of biopsies per man with clinically-detected prostate cancers.

This parameter has two components: (i) men with clinically detected prostate cancer may have had one or more previous negative biopsies; and (ii) men who had clinical symptoms with an associated negative biopsy but who were not clinically diagnosed with prostate cancer. However, limited data were available to calculate these components.

Using the Stockholm PSA and Biopsy Register, we extracted the number of men in Stockholm in 2012 diagnosed with prostate cancer with clinical symptoms; for those

Category	Unit	Unit cost [€, 2019]
PSA	Primary care (test sampling)	30
	PSA analysis	4
	GP primary care	0.2×131
S3M	Primary care (test sampling)	30
	PSA analysis	4
	S3M unit cost	196
	GP primary care	0.2×131
Biopsy	Prostate biopsy	103
	Prostate pathology	300
Assessment	Urology assessment	153
Prostatectomy	Surgery	6806
	Radiation therapy	0.25×8508
	Urology visit	2×153
	Nurse visit	2×103
Radiation therapy	Radiation therapy	8508
	Urology visit	2×153
	Nurse visit	2×103
Active surveillance - yearly	Urology visit	153
	PSA analysis	2×4
	Biopsy	0.5×403
Active surveillance - single MR	Used once for active surveillance	263
Post-treatment follow-up - yearly	PSA test sampling	30
	PSA analysis	4
	Telefollow-up by urologist	40
Cancer death	Care for spread disease	3×8521
	Drugs for spread disease	3×5785

S1 Table. Detailed costs for prostate cancer testing, diagnosis, management and treatment, Sweden [1, 2].

men, we found whether the men had any negative biopsies prior to their cancer diagnosis; we also assumed that a prostate cancer diagnosis had at least one biopsy. On average, these men had 1.24 biopsies at or before their prostate cancer diagnosis. We also extracted the number of men diagnosed with prostate cancer in 2012 and compared that number with the number of prostate biopsies for men not currently diagnosed with prostate cancer in 2012. This crude ratio was approximately 3 biopsies per prostate cancer diagnosis. Notably, these calculations are in the context of a population with moderate to high levels of PSA testing.

In summary, the number of biopsies associated with clinically detected prostate cancers is in the range of 1.24 to 3 biopsies per cancer. As an approximate estimate, we assumed 2 biopsies per clinically detected prostate cancer.

Sensitivity Analysis

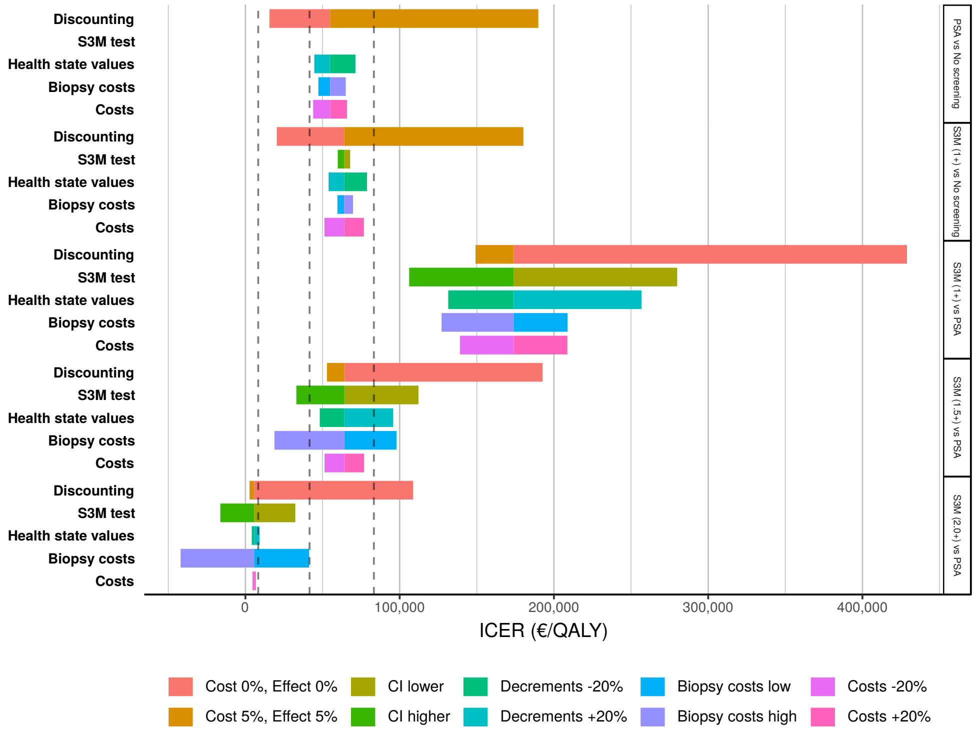

S1 Fig. One-way sensitivity analysis showing the effect of no and high discounting rates, the S3M test performance, high and low biopsy costs, 20% variation in all costs and HSV decrements on the cost-effectiveness. From the top the panels show the ICERs sensitivity for PSA screening compared with no screening, S3M screening compared with no screening, S3M screening compared with PSA screening as a reflex test at PSA 1, 1.5 and 2 ng/mL. From the left, the dashed lines show the limits for low (less than €8,300), moderate (€8,300–41,600), high (€41,600–83,300), and very high costs (over €83,300) for Sweden.

We performed a probabilistic sensitivity analysis quantifying the prediction uncertainty. Parameters from the natural history model were sampled using a multivariate normal distribution and covariance matrix from the model fit. All other parameters were assumed to be independent. The published confidence intervals from the published S3M test performance were sampled using a normal distribution. The costs for biopsies and urology assessments, which also indirectly affects the cost of active

surveillance, were handled separately, and sampled from a log-normal distribution €560 (95% CI 330-880). The other costs and the health state value decrements where sampled from triangular distributions with extremes ± 20%. The model was evaluated 500 times resulting in a distribution of outputs that can be graphed on the cost-effectiveness plane, and analysed. This may be represented using a cost-effectiveness acceptability curve.

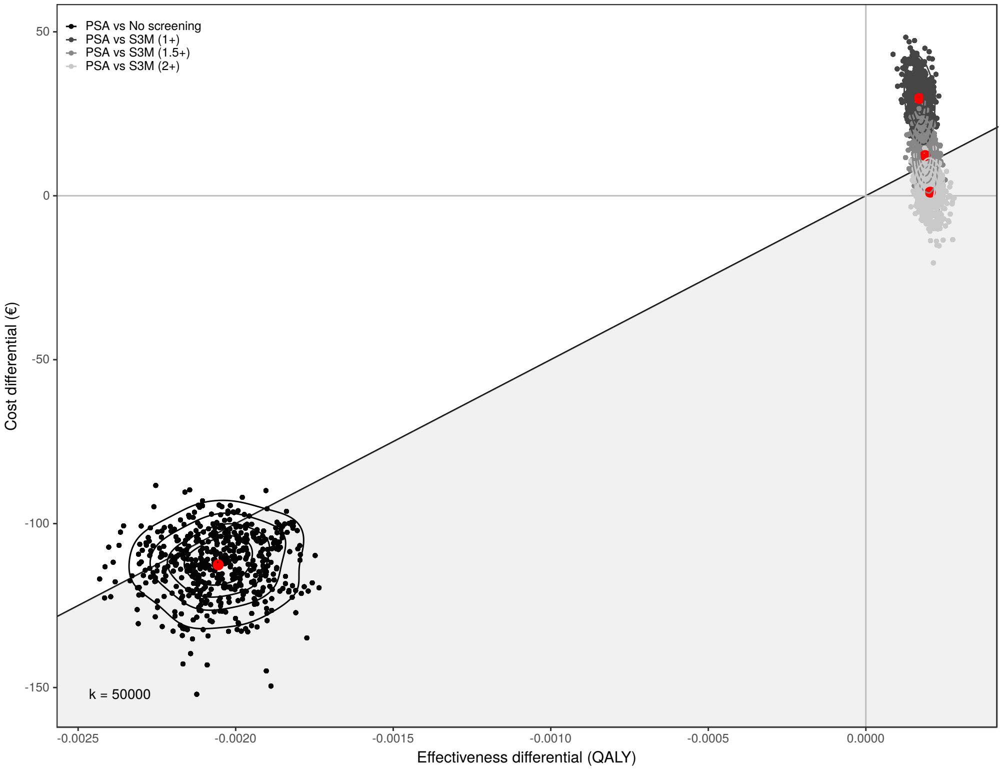

S2 Fig. Probabilistic sensitivity analysis for cost-effectiveness comparing no screening, PSA screening and S3M screening with reflex thresholds at 1 ng/mL, 1.5 ng/mL and 2 ng/mL. The effectiveness and costs are incremental relative to PSA screening and are discounted at 3% per annum. The red points show the distribution modes and the grey line shows a willingness to pay threshold at €50,000 per QALY.

The probabilistic sensitivity analysis represented on the cost-effectiveness plane comparing no screening, PSA screening and S3M screening with reflex thresholds at 1 ng/mL, 1.5 ng/mL and 2 ng/mL is shown in S2 Fig. The effectiveness and costs are incremental to PSA screening and discounted at 3% per annum. The cost reduction for no screening together with a 95% credible interval (CrI) was €112 (95% CrI 98–130). The cost increments for S3M screening with reflex thresholds at 1 ng/mL, 1.5 ng/mL and 2 ng/mL were €30 (95% CrI 19–41), €12 (95% CrI 3–21) and €1 (95% CrI -8–9), respectively.

The incremental reduction in effect for no screening was 0.0021 (95% CrI 0.0023–0.0018) QALYs relative to PSA screening. The effect increments for S3M screening with reflex thresholds at 1 ng/mL, 1.5 ng/mL and 2 ng/mL were 0.00017 (95% CrI 0.00013–0.00021), 0.00019 (95% CrI 0.00015–0.00022) and 0.00020 (95% CrI 0.00017–0.00024) QALYs, respectively.

Health outcomes before and after age 70 years

Health outcomes were represented as life-time measures in S3 Table. To represent the effect of screening on these outcomes, we stratified the health outcomes for effects before and after age 70 (S2 and S3 Tables). In outline, we see a substantial increase in prostate cancer incidence due to screening, with lower incidence in the screening interventions after age 70. We also observe a large increase in prostate cancer mortality in the group above 70 years of age.

Events before Age 70	No screening	PSA	S3M[a]	S3M[b]	S3M[c]	PSA-no screening	S3M[a]-PSA	S3M[b]-PSA	S3M[c]-PSA
**Outcomes per 10,000 men**						**Differences**			
Screening tests	0	35696	34875	34801	34732	35696	-820	-895	-963
Biopsies	523	6074	4046	3853	3663	5550	-2028	-2221	-2410
Negative biopsies	256	5430	3423	3236	3054	5173	-2006	-2194	-2376
Diagnosed cancers	267	645	623	618	610	378	-22	-27	-35
Screen-detected cancers	0	525	502	497	488	525	-23	-28	-37
Overdiagnosed cancers	0	118	107	104	100	118	-11	-14	-18
Prostate cancer death	28	21	21	21	21	-7	0	0	0

S2 Table. Predicted effects before age 70 for no screening, PSA screening and S3M screening at 4-year intervals for the ages 55 to 69 years. The effects are presented as clinical events per 10,000 men and the four right most columns shows the differences between the scenarios.

[a] S3M as a reflex test between 1 and 10 ng/mL [3].
[b] S3M as a reflex test between 1.5 and 10 ng/mL as per clinical practice [2].
[c] S3M as a reflex test between 2 and 10 ng/mL.

Events from Age 70 Outcomes per 10,000 men	No screening	PSA	S3M[a]	S3M[b]	S3M[c]	PSA-no screening	S3M[a]-PSA	S3M[b]-PSA	S3M[c]-PSA
						Differences			
Screening tests	0	108	68	64	60	108	-40	-44	-48
Biopsies	2266	1872	1857	1859	1862	-394	-15	-14	-10
Negative biopsies	1046	909	884	883	883	-137	-25	-26	-26
Diagnosed cancers	1220	964	973	976	978	-256	9	12	16
Screen-detected cancers	0	10	7	7	6	10	-3	-3	-3
Overdiagnosed cancers	0	3	2	2	2	3	-1	-1	-1
Prostate cancer death	474	409	412	412	414	-64	2	3	4

S3 Table. Predicted effects after age 70 for no screening, PSA screening and S3M screening at 4-year intervals for ages 55 to 69 years. Screening tests after the age of 70 are due to ongoing work-up originating in previous negative biopsies. The effects are presented as clinical events per 10,000 men and the four right most columns shows the differences between the scenarios.

[a] S3M as a reflex test between 1 and 10 ng/mL [3].
[b] S3M as a reflex test between 1.5 and 10 ng/mL as per clinical practice [2].
[c] S3M as a reflex test between 2 and 10 ng/mL.

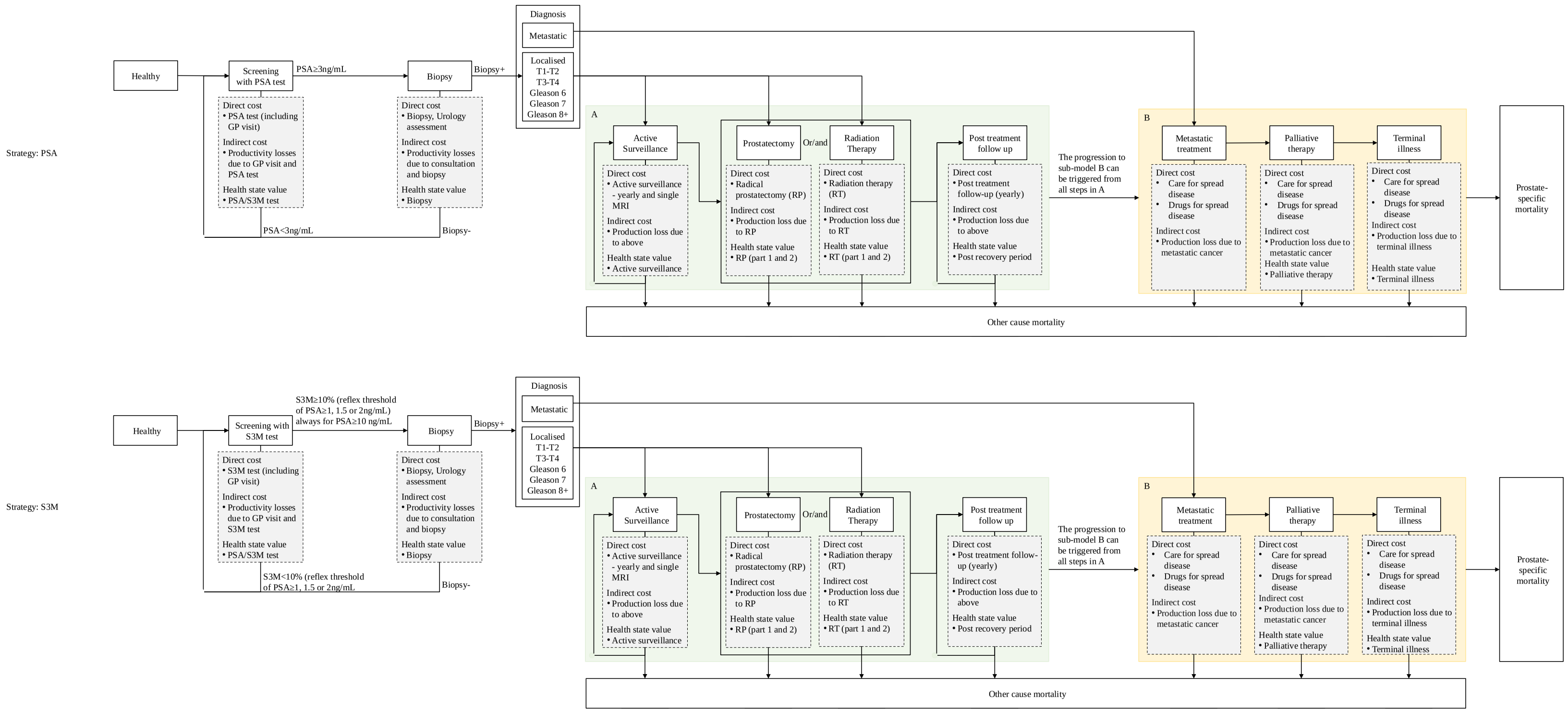

Sub-model A: Treatments for patients with localised prostate cancer
Sub-model B: Treatments for patients with metastatic cancer; Palliative therapy and Terminal care.
GP: General Practitioner; MRI: Magnetic Resonance Imaging; PSA: Prostate-Specific Antigen;
RP: Radical Prostatectomy; RT: Radiation Therapy; S3M: Stockholm3 test

S3 Fig. Detailed depiction of the health economic aspects of the model structure.

References

1. Socialstyrelsen. Screening för prostatacancer med PSA- prov. Hälsoekonomisk analys. Stockholm: Socialstyrelsen; 2018. Available from: https://www.socialstyrelsen.se/SiteCollectionDocuments/2018-2-13-halsoekonomisk-analys.pdf.

2. Karolinska Universitetslaboratoriet. Prostatacancertestet Stockholm-3; 2018. https://www.karolinska.se/KUL/Alla-anvisningar/Anvisning/10245.

3. Grönberg H, Adolfsson J, Aly M, Nordström T, Wiklund P, Brandberg Y, et al. Prostate cancer screening in men aged 50–69 years (STHLM3): a prospective population-based diagnostic study. The Lancet Oncology. 2015;16(16):1667 – 1676. doi:http://dx.doi.org/10.1016/S1470-2045(15)00361-7.
